# Multicentric Study on Enteric Protists Occurrence in Zoological Parks in Portugal

**DOI:** 10.3390/pathogens13100874

**Published:** 2024-10-06

**Authors:** João Mega, Rafaela Moreira, Guilherme Moreira, Ana Silva-Loureiro, Priscilla Gomes da Silva, Claudia Istrate, Sérgio Santos-Silva, Antonio Rivero-Juarez, David Carmena, João R. Mesquita

**Affiliations:** 1 ICBAS—School of Medicine and Biomedical Sciences, Porto University, 4050-313 Porto, Portugal; joaomega@outlook.com (J.M.); rafaelasimaomoreira@gmail.com (R.M.); gmoreiravet@gmail.com (G.M.); ana.mercedes.loureiro@gmail.com (A.S.-L.); up202002072@edu.icbas.up.pt (P.G.d.S.); up202110051@edu.icbas.up.pt (S.S.-S.); 2 Epidemiology Research Unit (EPIUnit), Public Health Institute, Porto University, 4050-600 Porto, Portugal; 3 Associate Laboratory for Integrative and Translational Research in Population Health (ITR), 4050-313 Porto, Portugal; 4 CIISA—Centre for Interdisciplinary Research in Animal Health, Faculty of Veterinary Medicine, University of Lisbon, 1300-477 Lisbon, Portugal; claudiaistratedvm@gmail.com; 5 Associate Laboratory for Animal and Veterinary Sciences (AL4AnimalS), 1300-477 Lisbon, Portugal; 6 Clinical Virology and Zoonosis, Infections Diseases Unit, Maimonides Biomedical Research Institute of Cordoba, Reina Sofía Hospital, Córdoba University, 14004 Córdoba, Spain; arjvet@gmail.com; 7 Infectious Diseases (CIBERINFEC), Health Institute Carlos III, 28029 Madrid, Spain; dacarmena@isciii.es; 8 Parasitology Reference and Research Laboratory, National Centre for Microbiology, 28220 Majadahonda, Spain

**Keywords:** *Balantioides coli*, *Blastocystis* sp., *Cryptosporidium* spp., *Eimeria* spp., *Giardia* spp., zoo, Portugal

## Abstract

Parasitic infections of the gastrointestinal tract of domestic animals play a major role in the transmission of disease, which in turn may result in financial and productive losses. Notwithstanding, studies on the burden and distribution of diarrheagenic protists in zoological gardens are still insufficient. Given the close animal–animal and animal–human interaction in these settings, Public Health concerns under the One Health context are raised. Using molecular detection tools and phylogenetic analysis, the goal of this study was to assess the occurrence of four potentially zoonotic protists—*Balantioides coli*, *Blastocystis* sp., *Cryptosporidium* spp. and *Giardia* spp.—in animals residing in zoological parks in Portugal. Occurrence of *Eimeria* spp. was also assessed because of its veterinary relevance. Although *Blastocystis* sp. represents most of the positive samples obtained (11.6%; 95% CI: 0.08–0.17), all parasites were detected (*B. coli* (2.9%; 95% CI: 0.01–0.06), and *Cryptosporidium* spp., *Eimeria* spp. and *Giardia* spp. presented the same prevalence (0.5%; 95% CI: 0.00–0.03)). We also describe the first molecular detection of *B. coli* in a collared peccary (*Tayassu tajacu*), of *Blastocystis* sp. in three different python species, and *G. muris* in a central bearded dragon (*Pogona vitticeps*), suggesting the broadening of the host range for these parasites.

## 1. Introduction

The global burden of infectious diseases is considerably increased by parasitic infections, as these illnesses play a major role in morbidity and mortality [1,2]. Furthermore, while parasitic infections primarily impact people in low-income countries [3,4,5], they also significantly contribute to illness in medium to high-income countries, where surveillance is often neglected [1].

Gastrointestinal (GI) protists have been widely reported in studies as being the causative agent of diarrhea in both animals [6,7,8,9,10,11,12,13,14] and humans [3,4,15,16,17]. Waterborne and foodborne transmission of GI protists results in water and foodborne outbreaks of gastrointestinal illnesses in human populations around the world, while close contact between infected individuals may cause sporadic infections [1].

In animals, infection by GI protists is known to reduce fertility, work ability, food intake, weight gain and milk production, significantly impacting animal health and causing considerable monetary losses, which is a documented indirect impact of protists in developing countries [6,7,8]. In heavily parasitized animals, infection may also lead to death [6,7,8].

Among the broad spectrum of GI protists capable of producing infections, *Balantioides coli*, *Blastocystis* sp., *Cryptosporidium* spp., *Eimeria* spp. and *Giardia* spp. are recognized as being potentially diarrheagenic [8,18].

*Balantioides coli* is a member of the Balantidiidae family [19]. Balantidiasis (symptomatic infection by this parasite) is a neglected disease which is common in rural communities where pig farming is practiced [20]. *Balantioides coli* is also the only ciliate known to infect humans [21], being capable of causing outbreaks [22]. The domestic pigs (*Sus scrofa domesticus*) and wild boars (*Sus scrofa*) are thought to be the main reservoir of infection [23]. Since disease in these hosts is usually asymptomatic, it becomes more difficult to avoid exposure for disease-free, naive populations of animals and humans [24]. Having no herd or population immunity, these individuals are more susceptible to infection [24]. Furthermore, 33 animal hosts have been reported to be infected by species of the genus *Balantioides*, including pigs, cattle, sheep, goats, camels, equids, human and non-human primates [23].

*Blastocystis* sp. is a prevalent anaerobic single-celled microeukaryote that infects the GI tract of hosts usually transmitted by the fecal–oral route [25]. As of the time of writing, 42 different subtypes have been suggested [26,27,28]. Infection is possible in a variety of hosts, ranging from companion animals (dogs, cats), livestock animals (domestic pigs, sheep, goats, cows and poultry), wild birds, mammals, non-human primates and humans [29]. Furthermore, the zoonotic potential of this protist has been recognized, as highlighted by evidence gathered in Spain and UK [30,31]. The possibility of reverse zoonosis has also been suggested, namely for domesticated animals [31,32,33,34,35]. Infection in humans is often asymptomatic but there are also reports linking *Blastocystis* sp. with GI (e.g., diarrhea, abdominal pain, flatulence, bloating and constipation) and extra-intestinal (e.g., urticaria, splenic cysts and Hashimoto’s thyroiditis) disease [36,37,38]. Considering that *Blastocystis* sp. is also commonly found in asymptomatic individuals [38], its pathogenic potential and clinical significance remain controversial [39,40]. Nevertheless, the elimination of *Blastocystis* sp. in these patients coincided with the disappearance of non-GI symptoms after treatment with metronidazole and/or paromomycin in cases of urticaria and Hashimoto’s thyroiditis [36,37,40,41].

Over 44 *Cryptosporidium* species and 120 genotypes of uncertain taxonomic status exhibiting variable degrees of host range and specificity have been identified to date [42], including the wide-ranging, zoonotic *Cryptosporidium parvum* and the anthroponotic *Cryptosporidium hominis* [43]. The infection causes self-limiting diarrhea and other clinical symptoms in immunocompetent individuals but can be life threatening if hosts are immunocompromised [42]. Additionally, it can also infect a wide range of vertebrate hosts [44]. Parasites of this genus are found in more than 150 mammalian species, with the order Rodentia (the most abundant and diverse order of mammals) also being reported as *Cryptosporidium* spp. reservoirs [45].

*Eimeria*, a genus in the Coccidia family, typically inhabits the host’s intestine. It has a strictly host-specific nature and follows a monoxenous life cycle [46]. The genus is estimated to comprise a large number of species [46]. These obligate intracellular parasites replicate in the intestines of a permanent host, undergoing successive rounds of asexual and sexual reproduction resulting in oocysts that are shed into the environment [47]. Regardless of individual species presenting remarkable host specificity, the genus has a broad range of hosts, being capable of infecting members of all vertebrate classes [46]. This protozoan does not raise zoonotic concerns, as it is not capable of infecting humans [46]. Eimeiriosis affects livestock animals such as poultry and ruminants (cattle, sheep and goats), leading to significant losses in the animal industry [8].

*Giardia* spp. are flagellated protozoans that parasitize the small intestines of mammals, birds, reptiles and amphibians [42]. Nine *Giardia* species are currently recognized [42]. Of them, *G. duodenalis* (the only species infective to humans) is categorized as a species complex consisting of eight distinct genetic variants or assemblages (A to H) [48,49]. *Giardia duodenalis* is the cause of giardiasis in humans and in most mammals [50,51], therefore being considered a zoonotic disease [52]. Infection typically results in clinical illness marked by diarrhea, abdominal cramps, bloating, weight loss and malabsorption [52]. Both developing and high-income countries are affected by the illness, with higher prevalences in developing countries [51,53]. *Giardia duodenalis* is also a widespread enteric parasite of domestic animals, including livestock, dogs and cats, as well as wild animals [53,54,55].

These protists were selected for surveillance due to the zoonotic potential and their role as water and foodborne parasites in the case of *B. coli*, *Blastocystis* sp., *Cryptosporidium* spp. and *Giardia* spp., while the veterinary relevance and lack of epidemiological data in these populations was the reason for *Eimeria* spp. screening. The aim of this work was to evaluate the spectrum of potential hosts for protists covered and evaluate the prevalence and molecular diversity of strains circulating in Portugal.

## 2. Materials and Methods

### 2.1. Study Design and Sample Collection

**Figure 1:**
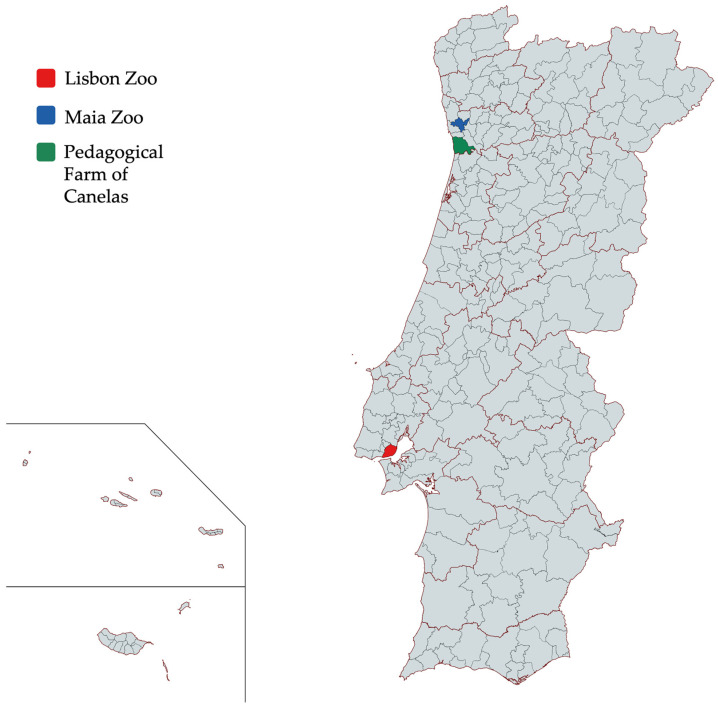
Location of the three Zoological Parks of Portugal considered for this study. Location of Lisbon Zoo is shaded in red, location of Maia Zoo is shaded in blue and location of Pedagogical Farm of Canelas is shaded in green.

This study screened 207 individual fecal samples from three different Zoological Gardens (Lisbon Zoo, Maia Zoo and Pedagogical Farm of Canelas), where animals were kept for educational, conservation and recreational purposes. Sampling was performed on animals belonging to 53 families. A total of 20 families were surveyed in Lisbon Zoo, 39 in Maia Zoo, while only 10 families were surveyed in the Pedagogical Farm of Canelas. Further information about distribution of individuals by family and discrimination of families represented in each zoological park is included in the Supplementary Materials (Tables S1–S4 and Figure S1). Locations of the three zoos considered in this study are shown in Figure 1.

Fecal samples were collected fresh from the ground after fecal excretion was confirmed. The vast majority of samples collected were formed fecal samples; only one sample from an otter (*Lutra lutra*) in the Maia Zoo presented diarrhea.

### 2.2. DNA Extraction and Purification

Following collection, fecal samples were immediately kept at −20 °C until aliquots were thawed for DNA extraction. PBS (phosphate-buffered saline) (pH = 7.2) was used to prepare 10% fecal suspensions, which were then centrifuged at 8000× *g* for 5 min. After centrifugation, 140 μL of cleared supernatants were used to extract and purify genomic DNA using the QIAamp DNA Stool Mini Kit (Qiagen, Hilden, Germany). The extraction procedure was completed using a QIAcube^®^ automated platform (Qiagen, Hilden, Germany). Eluted DNA (100 μL) was kept in DNase-free water at −80 °C until molecular testing.

### 2.3. Molecular Detection

To detect *B. coli*, a PCR assay targeting the complete ITS1–5.8s-rRNA–ITS2 region and the last 117 bp at the 3′-end of the small subunit ribosomal RNA (*SSU*-rRNA) gene of the parasite (400 bp) was used with the primer set B5D/RD5 as proposed by Ponce-Gordo et al. [56]. For the identification of *Blastocystis* sp., an endpoint PCR approach that targets a 600-bp region of the *SSU*-rRNA gene with the pan-*Blastocystis* primer set RD5/BhRdr was performed as previously described [57]. For the identification of *Cryptosporidium* spp., a nested-PCR assay was used to amplify a 587-bp fragment of the *SSU*-rRNA gene of the parasite using the set of primers CR-P1/CRP2 (first round) and CR-P3/CPB-DIAGR (second round), as previously described [58]. After identification of one positive sample as *C. ubiquitum,* subtyping was carried by a nested-PCR assay for the *gp60* gene of this parasite using primers Ubi-18S-F1/Ubi-18S-R1 and Ubi-18S-F2/Ubi-18S-R2, as described [59]. For the detection of *Eimeria* sp., a semi-nested-PCR was used to amplify a 420-bp fragment of the *SSU*-rRNA gene of the parasite with the primer set Ei18sF/Ei18sR and Ei18sF/ER10 [60]. For the detection of *Giardia* spp., a nested-PCR assay was used to amplify a 293-bp region of the *SSU* rRNA of the parasite with the primer pairs RH11 derivatives/Gia2150c and RH11 derivatives/RH4 derivatives, as previously described [61]. Table 1 outlines primers used, while PCR cycling conditions are summarized in the Supplementary Materials (Table S5). Only molecular techniques were used to detect the presence of parasites.

### 2.4. Electrophoresis

Following PCR amplification, identification of positive samples was performed by electrophoresis on 1.5% agarose gels. These gels were stained with Xpert Green Safe DNA gel dye (GRiSP^®^, Porto, Portugal). Electrophoresis had a duration of 30 min at a constant voltage of 120 V.

### 2.5. Sanger Sequencing and Phylogeny

Amplicons presumed positive were purified using the GRS PCR & Gel Band Purification Kit (Grisp^®^) and submitted for Sanger bidirectional sequencing using the internal primer sets described above. Raw sequences were edited with BioEdit Sequence Alignment Editor v7.1.9 software package, version 2.1. Chromatograms were visually inspected for the presence of ambiguous (double peak) positions. Obtained consensus sequences were further compared with those available in the NCBI (GenBank) nucleotide database (http://blast.ncbi.nlm.nih.gov/Blast) for confirmation, accessed on 25 June 2024.

All sequences obtained in this study were deposited in GenBank under accession numbers OM349074–OM349076, OR987597, OR987497 and OR987498 (*B. coli*); OR987545–OR987552, OR989903–OR989916, OR977104 and OR987485 (*Blastocystis* sp.); OR987500 (*SSU*-rRNA) and PP579943 (*gp60*) (*Cryptosporidium* spp.), OR987677 (*Eimeria* spp.) and OR991119 (*Giardia* spp.).

MEGA version X software was used to infer phylogenetic trees assessing sequence diversity within and between groups of sequences [62,63]. The analysis included sequences generated in the present study and curated sequences retrieved from GenBank for comparison and referencing purposes. The maximum-likelihood (ML) method [62,63] was used to assess evolutionary relationships and create the phylogenetic trees. The Tamura 3-parameter model (*B. coli*, *SSU*-rRNA and *gp60 Cryptosporidium* spp. genes, *Eimeria* spp. and *Giardia* spp.) and the General Time Reversible model (*Blastocystis* sp.), which MEGA X determined to be the best substitution model [62], was used to estimate the ML bootstrap values using 1000 replicates.

Prevalences of GI protists in stools were calculated based on the proportion of the number of positive samples to the total number of samples examined with a 95% confidence interval (95% CI).

## 3. Results

Collection and screening of the overall 207 fecal samples from the three sites, namely Lisbon Zoo (*n* = 81), Maia Zoo (*n* = 76) and Pedagogical Farm of Canelas (*n* = 50), resulted in 33 samples positive for at least one of the diarrheagenic protist species screened for in this study, resulting in a prevalence of 15.94% (33/207, 95% CI: 11.24–21.65). No co-infections were found. *Balantioides coli* and *Blastocystis* sp. were the most prevailing parasites, but all protists surveyed tested positive in at least one individual. An overview of occurrence is provided in Table 2. Results outlined by each zoological garden included in this work are described in detail in the Supplementary Materials. In this work, isolates are given codes for site of collection: ZL-code for sequences retrieved from the Lisbon Zoo; ZM-code for sequences retrieved from the Maia Zoo; Q-code for sequences retrieved from the Pedagogical Farm of Canelas.

### 3.1. Balantioides coli

Prevalence of *Balantioides coli* was 2.9%, with six positive results in the 207 samples tested (95% CI: 0.01–0.06), being the second most prevalent parasite in this study. Presence of this protozoa was detected in all zoological gardens covered. Sequence similarity analysis within positive samples retrieved of *B. coli* showed that sequences shared 91.64–99.75% identity between them.

**Figure 2:**
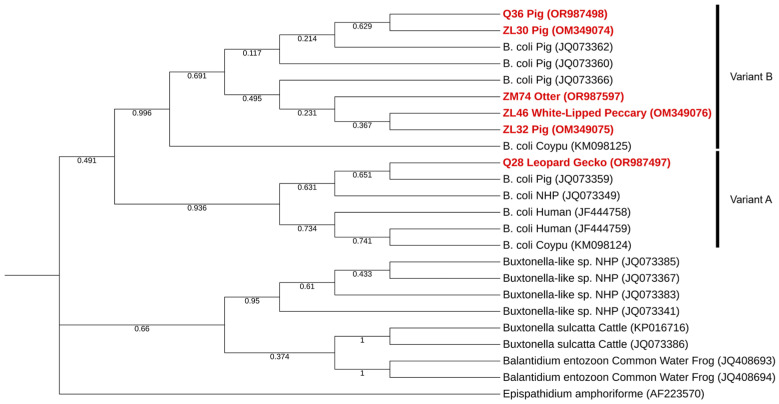
Phylogenetic tree inferred using the MEGA X maximum likelihood method (Tamura 3-parameter model) and the Interactive Tree Of Life (iTOL) based on 24 nucleotide sequences including those generated in this study (highlighted in bold and shaded in red). *Balantidium entozoon* is a member of the same family (Balantidiidae), while *Buxtonella sulcatta* (*Buxtonella*-like sp. as well) is a member of the Pycnotrichidae, family of the same order (Vestibuliferida) of Balantidiidae. *Epispathidium amphoriforme* was used as an outgroup.

Phylogenetic analysis of *B. coli* showed that only one sequence collected at Pedagogical Farm of Canelas corresponded to genetic variant A, while all sequences obtained from the Lisbon Zoo, one from Pedagogical Farm of Canelas and the only sequence retrieved at the Maia Zoo was found to belong within a well-supported cluster with other *B. coli* sequences corresponding to genetic variant B (Figure 2). A summary of *B. coli* positive samples is shown in Table 3, alongside single nucleotide polymorphisms (SNPs) between sequences obtained and reference sequences.

### 3.2. Blastocystis sp.

*Blastocystis* sp. was found to be the most prevalent parasite in this work, as 24 positive samples were detected. This protist showed a prevalence of 11.6% (95% CI: 0.08–0.17). As was the case of *B. coli*, positive results were found in all zoological gardens. *Blastocystis* sp. sequences shared 77.55–100% identity between them.

**Figure 3:**
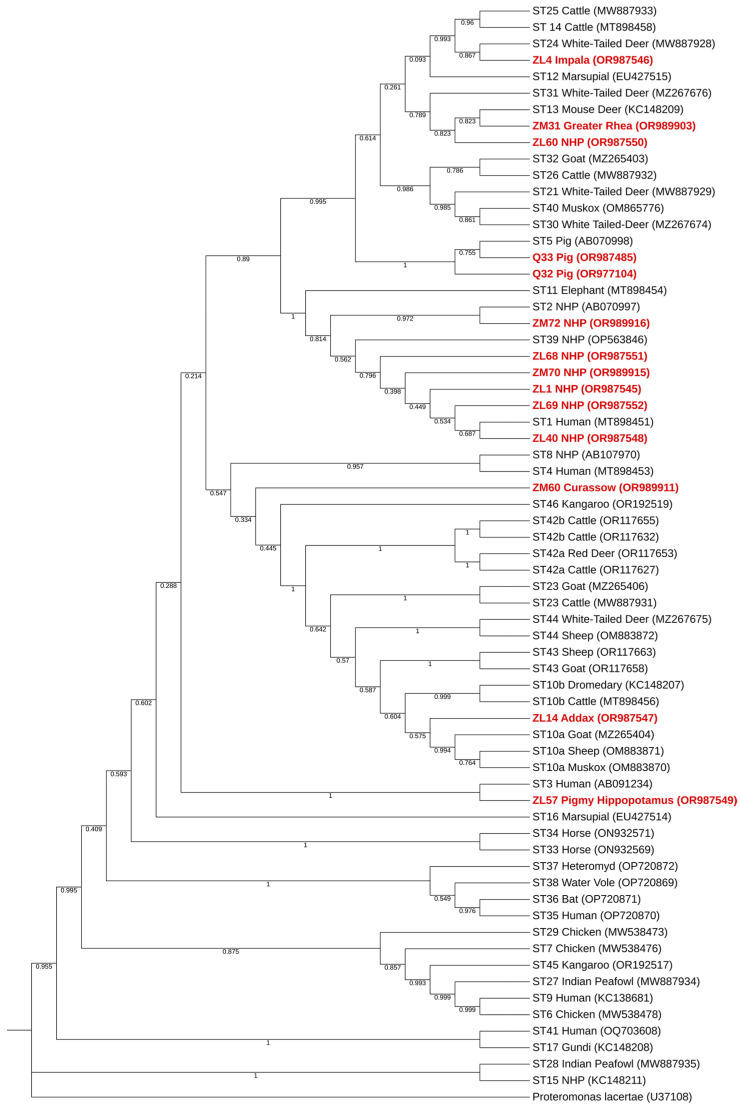
Phylogenetic analysis inferred using the MEGA X maximum likelihood method (General Time Reversible model) and the Interactive Tree Of Life (iTOL) based on 67 nucleotide sequences of *Blastocystis* sp., including those generated in this study that were obtained from avian and mammalian sources (highlighted in bold and shaded in red). A *Proteromonas lacertae* sequence is used as an outgroup.

Phylogenetic analysis of *Blastocystis* sp. showed that all samples cluster with recognized reference sequences of the protist (Figure 3). Sequences ZM33, ZM45-ZM49, ZM52, ZM63, ZM64 and ZM67 were obtained from reptilian sources, and STs available are only valid for sequences obtained from mammalian and avian sources; as such these sequences are not shown in this phylogenetic tree. Most did not cluster with any previously established subtype, as expected, but all appear within the *Blastocystis* sp. clade. Sequence ZM60 did not cluster with any specific subtype but falls within the *Blastocystis* sp. clade. Positive results of *Blastocystis* sp. and single nucleotide polymorphisms (SNPs) between sequences obtained and reference sequences are summarized in Table 4.

### 3.3. Cryptosporidium spp.

Only one positive sample of *Cryptosporidium* spp. was detected, resulting in a prevalence of 0.5% (95% CI: 0.00–0.03). This positive sample (Q16) was detected in the Pedagogical Farm of Canelas in a horse (*Equus ferus caballus*) and given the Genbank ID-OR987500.

**Figure 4:**
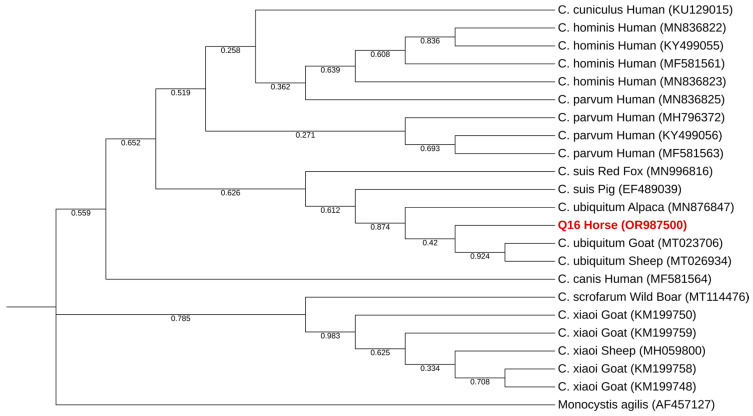
Phylogenetic analysis using the MEGA X maximum likelihood method (Tamura 3-parameter model) and the Interactive Tree Of Life (iTOL) based on 22 *SSU*-rRNA sequences of *Cryptosporidium* spp., including the one generated in this study (highlighted in bold and shaded in red).

Phylogenetic analysis of the only *Cryptosporidium* spp. sequence obtained identified it as *C. ubiquitum*. The sequence falls in a well-supported cluster of sequences previously classified as such (Figure 4). Single nucleotide polymorphisms (SNPs) between the sequence obtained and a reference sequence are presented in Table 5.

**Figure 5:**
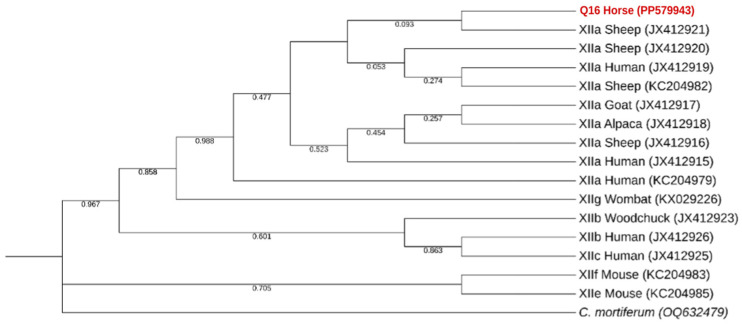
Phylogenetic analysis using the MEGA X maximum likelihood method (Tamura 3-parameter model) and the Interactive Tree Of Life (iTOL) based on 16 *gp60* sequences of *Cryptosporidium ubiquitum*, including the one generated in this study (highlighted in bold and shaded in red) and a further strain of the *gp60* gene of *Cryptosporidium mortiferum* that serves as the outgroup.

To confirm the result obtained at the *SSU*-rRNA gene marker and to identify the genotype subtype of the *C. ubiquitum* isolate identified, we re-assessed this sample at the 60 kDa glycoprotein (*gp60*) marker. Sequence analyses revealed that it belonged to genotype family XIIa (Figure 5).

### 3.4. Eimeria spp.

*Eimeria* spp. was also detected in only one sample, resulting in a prevalence of 0.5% (95% CI: 0.00–0.03). This positive sample (ZM26) was detected in the Maia Zoo in a peacock (*Pavo cristatus*) and given the Genbank ID-OR987677.

**Figure 6:**
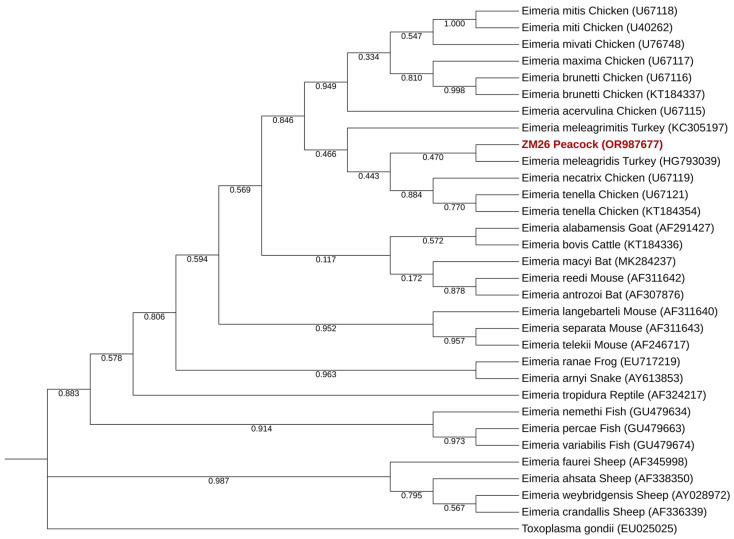
Phylogenetic tree inferred using the MEGA X maximum likelihood method (Tamura 3-parameter model) and the Interactive Tree Of Life (iTOL) based on 32 sequences. Of those, 30 are recognized sequences of *Eimeria* spp., one was generated in this study (highlighted in bold and shaded in red) and the last serves as the outgroup (*Toxoplasma gondii*).

Phylogenetic analysis of *Eimeria* spp. revealed that the sequence obtained falls in a well-defined cluster for the genus and suggests classification as *E. meleagridis* (Figure 6). Single nucleotide polymorphisms (SNPs) between the sequence obtained and a reference sequence are presented in Table 6.

### 3.5. Giardia spp.

Detection of *Giardia* spp. was restricted to only one sample, as the two previous protists represented, consequently resulting in the same prevalence of 0.5% (95% CI: 0.00–0.03). This positive sample (ZM40) was detected in the Maia Zoo in a bearded central dragon (*Pogona vitticeps*) and given the Genbank ID-OR991119.

**Figure 7:**
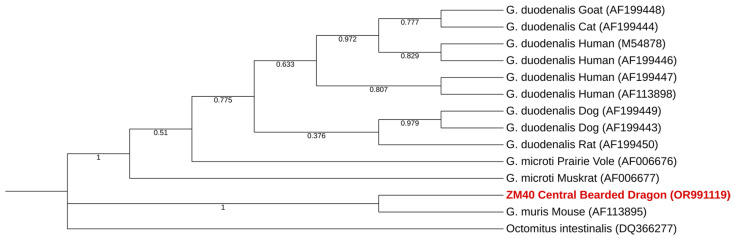
Phylogenetic tree ascertained using the MEGA X maximum likelihood method (Tamura 3-parameter model) and the Interactive Tree Of Life (iTOL) based on 14 nucleotide sequences including those generated in this study (highlighted in bold and shaded in red). *Octomitus intestinalis* belongs to the genus *Octomitus*, which is also part of the subfamily Giardiinae (alongside the genus *Giardia*).

Phylogenetic analysis of *Giardia* spp. showed that the only positive sequence in this study clustered with a sequence of *Giardia muris* (Figure 7). Single nucleotide polymorphisms (SNPs) between the sequence obtained and a reference sequence are presented in Table 7.

### 3.6. Clinical Impact of Infections

Apart from one, all individuals surveyed presented as asymptomatic. The only sample that presented diarrhea was from an otter (*Lutra lutra*) residing in the Maia Zoo that tested positive for *B. coli*. At the time of collection, diarrhea was the only symptom presented and the individual was quarantined.

## 4. Discussion

Studies have reported high parasitic infection rates in zoo animals, with protozoa being among the most frequent parasites of animals in these settings [64,65,66,67,68,69,70]. When living free in the wild, animals occupy broad areas and are exposed to many parasites such as protozoa, naturally acquiring resistance to these parasites [64,69]. In zoo enclosures, animals are kept in confined places and therefore are more prone to intimate contact which can facilitate transmission. Also, since little movement is allowed when in captivity, animals are less frequently exposed to a variety of these agents, which can lower their immunological resistance to such illnesses [64,69]. In addition to that, the immunity of captive animals to parasite illnesses can be boosted (or lowered) depending on their diet, with stressful conditions brought on by the captivity also reducing the animal’s resistance to parasite infections [64,68,71].

Epidemiological studies using molecular detection already exist in European zoos. In Córdoba Zoo Conservation Centre (Spain), a prevalence of 45.1% of *Blastocystis* sp., 21.6% of *G. duodenalis* and 3.9% of *B. coli* was found in 51 fecal samples of non-human primates (NHPs) [72]. Another study, conducted in several zoological gardens in Europe (one in France, one in Germany and four in Spain), also only with samples of NHPs, showed a prevalence of 20.3% for *Blastocystis* sp., 18.1% for *G. duodenalis*, 1.5% for *B. coli* and 0.9% for *Cryptosporidium* spp. in 454 fecal samples [30]. Comparisons with the studies presented might be skewed as both previous studies only focus on NHP samples; however, parallelisms can be drawn. In both and in our work as well, *Blastocystis* sp. is the protist with the higher prevalence, and *B. coli* and *Cryptosporidium* spp. frequencies are similar between studies. Furthermore, prevalence found in the Maia Zoo for *Blastocystis* sp. is very similar with the prevalence found in the multicentric study (in France, Germany and Spain).

Another multicentric report of infection in NHPs (non-human primates) (France) shows a rate of infection by protozoa (including *B. coli* and *Giardia duodenalis*) of 47.3% in 252 samples tested [69]. However, caution should be taken when comparing to this latter study, as this was a coprological survey by direct examination; hence sensitivities and specificities are distinct to those obtained by molecular detection, demonstrated by the inability to identify a significant portion of the protozoa found.

Of the 33 individuals that tested positive for any of the protists covered in this work, only one presented symptoms. An otter (*Lutra lutra*) residing in Maia Zoo tested positive for *B. coli*, had diarrhea and was quarantined. This animal tested positive for *B. coli*, but infection with other enteric agents not covered in this work cannot be discarded, so correlation between this infection and the symptom shown is not possible. The majority of animals carrying enteric parasites were apparently healthy and produced formed fecal material, suggesting absence of gastrointestinal manifestations. Under these circumstances, infected animals can act as inadvertent spreaders of the infections to other captive animals or even humans (as vets and zookeepers have direct contact with these animals and are responsible for the disposal of excrement and overall maintenance of enclosures). This highlights the need for proper management practices that should be implemented to minimize transmission risk, including adequate excreta disposal and extra care for personal hygiene habits (by zookeepers and vets), food quality and isolation of animals that present symptoms. Alongside these management practices, regular scheduled screenings of parasites and other infectious agents could be valuable to prevention and an asset for public health, especially in the context presented here, where most infected animals are asymptomatic.

In the present study, *B. coli* was detected in two Vietnamese pot-bellied pigs and a domestic pig (*Sus scrofa domesticus*), in a collared peccary (*Tayassu tajacu*), in a common otter (*Lutra lutra*) and a leopard gecko (*Eublepharis macularis*). Phylogenetic analysis of samples retrieved shows that all samples fall within a well-supported cluster with reference sequences of *B. coli*, with only one (detected in the leopard gecko in the Pedagogical Farm of Canelas) clustering with sequences corresponding to genetic variant A, while all the others clustered with sequences corresponding to genetic variant B. Although only variant A was confirmed to be present in human samples, there is no indication that variant B is incapable of zoonotic transmission [73]; therefore risk of transmission to humans might still be present.

To the best of the authors’ knowledge, potentially novel hosts of *B. coli* were detected in this work. Detection of *B. coli* in a collared peccary (*Tayassu tajacu*) in the Lisbon Zoo is one of these cases. The two other cases of infection by the same parasite detected in this zoo were found in well-known reservoirs: two pot-bellied pigs (*Sus scrofa domesticus*). It is important to note that all three positive individuals were born in the Lisbon Zoo and that the *Sus scrofa domesticus* and *Pecary tajacu* individuals are located in opposite areas from each other; therefore they have never been in close contact, excluding the possibility of direct transmission. Fruits and vegetables could have been the source of transmission in this case since they are part of the diet of both species in the zoo. Interestingly, sequence similarity analysis within these three *B. coli* positive samples retrieved shows that sequences shared 98.77–99.60% identity between them, which suggests distinct origins.

The collared peccary (*Tayassu tajacu*), a member of the Tayassuidae family, and the Vietnamese pot-bellied pig (*Sus scrofa domesticus*), a member of the Suidae family, both share morphological similarities and are members of the Suina suborder, order Artiodactyla [74]. Notwithstanding, they can interact with similar parasitic species, with diarrheic protists being one of the several parasites that infect swine and are known to significantly increase morbidity in wild boars and domestic pigs, as is the case of *B. coli* [75]. Three species have currently been identified as belonging to the family Tayassuidae: *Tayassu pecari* (white-lipped peccary), *Catagonus wagneri* (Chacoan peccary or tagua) and *Tayassu tajacu* (collared peccary). Because pigs are the most common animal infected with *B. coli*, and considering their close phylogenetic relationship with peccaries, it makes them a probable species to be infected by this protozoon [76]. Interestingly, there is a report of a single description from microscopical identification of a ciliate indistinguishable from *B. coli*, although with no molecular confirmation, in a collared peccary in Texas (USA) in 1970 [77]. Our work seems to confirm this result, being the first molecular description of this parasite in a collared peccary.

Infection by *Blastocystis* sp. was found in 24 individuals from 12 distinct families (Agamidae, Bovidae, Cercopithecidae, Cracidae, Gherrosauridae, Hippopotamidae, Hominidae, Iguanidae, Pithonidae, Rheidae, Scincidae and Suidae). Phylogenetic analysis shows clustering with reference sequences previously recognized as *Blastocystis* sp. Isolates ZL1, ZL40, ZL57, ZL68, ZL69, ZM67, ZM72, Q32 and Q33 clustered with reference sequences of recognized *Blastocystis* sp. zoonotic STs (ST1, ST2, ST3, ST5, ST7, ST8, ST10a) [78]. This fact in combination with evidence of zoonotic transmissions [30,72] raises concerns about possible zoonotic transmission of *Blastocystis* sp. happening in zoological gardens in Portugal.

Isolates ZM33, ZM45-ZM49, ZM63 and ZM64, obtained from reptile hosts, did not cluster with any previously established subtype but fell well within the protist clade. The classification system based on STs for *Blastocystis* sp. was constructed for avian and mammalian species, explaining why classification of isolates from reptilian sources might not always work. Isolate ZM60 (obtained from *Crax rubra*, an avian source) also did not cluster with any specific subtype.

Hosts not previously described for *Blastocystis* sp. were also potentially found, as a Darwin carpet python (*Morelia spiglota variegate*), a royal python (*Python regius*) and three Burmese pythons (*Python bivittatus*) all showed positive samples. Notwithstanding, the presence of this protist genus in other types of pythons and other reptiles has previously been reported [79], with *Blastocystis pythoni* serving as an example of a species of this parasite first described in reptiles [80]. However, as far as the authors know, none of the python and reptile species mentioned above appear to have been reported as hosts.

Only one positive sample of *Cryptosporidium* spp. was found, in a horse (*Equus ferus caballus*) residing at the Pedagogical Farm of Canelas. This parasite was detected in neither of the other locations present in the study. Phylogenetic analysis indicates that *Cryptosporidium ubiquitum* was detected. Afterward, this classification was confirmed by a positive result for amplification of the *gp60* gene of *Cryptosporidium ubiquitum*. This also permitted classification of the isolate found as XIIa subtype. As this subtype is primarily described in domestic and wild ungulates, including sheep, goat, yak, horse, alpaca and buffalo, our result aligns with the research available [59]. Although most human cases of cryptosporidiosis are primarily caused by anthroponotic *C. hominis* and zoonotic *C. parvum* (and, to a much lesser extent, by host-adapted *C. meleagridis*, *C. felis* and *C. canis*) [81], *C. ubiquitum* is an emerging zoonotic pathogen in humans, as it was responsible for more cases of human cryptosporidiosis in the UK than *C. canis*, in samples collected between 2000 and 2008 [82]. This suggests the need for more surveillance for otherwise neglected species of the genus *Cryptosporidium*.

*Eimeria* spp. was also detected in this work, in a peacock (*Pavo cristatus*) residing at the Maia Zoo. Phylogenetic analysis confirmed the sequence retrieved as belonging to the *Eimeria* genus, showing higher similarity with *E. meleagridis* (which was expected, as our sequence was obtained from an avian source and this species of *Eimeria* is common in birds). Unequivocal identification of the isolate found is difficult, as *Eimeria* spp. is a genus that comprises a large number of species, with genetic variation and sexual differentiation in both endogenous and exogenous coccidial stages, presenting a challenge for accurate classification [83,84,85]. For these reasons, classification of avian *Eimeria* regarding species usually requires phylogeny using the whole genome, while phylogeny using the *SSU*-rRNA normally only confirms genus [86]. As such, this result should be considered with caution.

*Giardia* spp. was found in the Maia Zoo in a central bearded dragon (*Pogona vitticeps*). Phylogenetic analysis shows that the sequence obtained belongs to the *Giardia* genus and indicates that *Giardia muris* was detected. The usual hosts for *G. muris* are rodents [53], and reports of *Giardia* spp. in reptiles are scarce. To the best of the authors’ knowledge, *Pogona vitticeps* is not previously described as a host of *Giardia muris*. Despite the abovementioned, caution should be taken when interpreting these results. Detection of protist DNA in stools might somehow be the result of mechanical passage through the intestines of the animals and not be necessarily correlated to enteric replication.

The present survey has some limitations. Firstly, sample size is limited by the number of individuals in the zoological parks mentioned. Also, except for *B. coli* and *Blastocystis* sp., the number of genotyped positive samples is diminutive (for *Cryptosporidium* spp., *Eimeria* spp. and *Giardia* spp., as only one positive sample was found for each). Consequently, with a limited sample size and diminutive genotyped positive samples for three of the five protists surveyed, results might not be representative for other captive wild animal populations. Furthermore, Sanger sequencing is not suited for detection of co-infections (infection of multiple STs of *Blastocystis* are an example) in the same sample. Another limitation is that no information about the occurrence and directionality of zoonotic transmission events is provided here, as no human samples could be retrieved.

## 5. Conclusions

In Summary, all parasites covered in this work were confirmed to be in circulation in Portuguese Zoological Gardens. The data here presented points to *Blastocystis* sp. as being the most represented parasite, and phylogenetic analysis of sequences obtained has also shown that, in these facilities, the majority of the *Blastocystis* sp. strains circulating are zoonotic, raising concerns. Furthermore, the vast majority of individuals were asymptomatic. These individuals that do not acquire the disease may operate as transmission sources, potentially being of significance to public health. Surveillance of urban areas using zoos as a detection tool of variants circulating might be valuable because it inherently presents a wide range of potential hosts in animals that have close contact with human populations.

This study also reports novel hosts, as it describes for the first time using molecular detection tools the presence of *B. coli* infection in a *Tayassu tajacu* individual. This is also the first report of *Blastocystis* sp. infection in three reptilian species (*Morelia spiglota variegate*, *Python regius* and *Python bivittatus*) and the presence of *G. muris* in *Pogona vitticeps*. These findings emphasize the need for documenting similar findings on hosts and parasitism origin for other animals in captivity.

## Figures and Tables

**Table 1:** Oligonucleotides used for the molecular identification and characterization of *Balantioides coli*, *Blastocystis* sp., *Cryptosporidium* spp, *Eimeria* spp. and *Giardia* spp. in the present study.

Target Organism	Locus	Primer	Sequence (5′-3′)	Reference
*Balantioides coli*	ITS region and the last 117 bp at the 3′ end of the *ssu*-rRNA	B5D B5RC	5′–GCTCCTACCGATACCGGGT–3′ 5′–ATATGCTTAAGTTCAGCGGG–3′	[56]
*Blastocystis* sp.	600 bp region of the *ssu*-rRNA gene	RD5 BhRdr	5′–ATCTGGTTGATCCTGCCAGT–3′ 5′–GAGCTTTTTAACTGCAACAACG–3′	[57]
*Cryptosporidium* spp.–*SSU-rRNA*	587 bp region of the *ssu*-rRNA gene	CR-P1 CRP2 CR-P3 CPB-DIAGR	5′–CAGGGAGGTAGTGACAAGAA–3′ 5′–TCAGCCTTGCGACCATACTC–3′ 5′–ATTGGAGGGCAAGTCTGGTG–3′ 5′–TAAGGTGCTGAAGG AGTAAGG–3′	[58]
*Cryptosporidium* spp.–*gp60*	948 bp region of the *gp60* gene	Ubi-18S-F1 Ubi-18S-R1 Ubi-18S-F2 Ubi-18S-R2	5′–TTTACCCACACATCTGTAGCGTCG–3′ 5′–ACGGACGGAATGATGTATCTGA–3′ 5′–ATAGGTGATAATTAGTCAGTCTTTAAT–3′ 5′–TCCAAAAGCGGCTGAGTCAGCATC–3′	[59]
*Eimeria* spp.	420 bp region of the *ssu*-rRNA gene	Ei18sF Ei18sR ER10	5′–CCCAATGAAAACAGYTTCGAGG–3′ 5′–AAACCCCCTACTGTCGTTCTTG–3′ 5′–GCCCCCAACTGTCCCTATTA–3′	[60]
*Giardia* spp.	293 bp region of the *ssu*-rRNA gene	RH11-derivates Gia2150c RH4–derivates	Equal mix of 5′–CATCCGGTCGATCCTGCC–3′ and 5′–CATCCGGTTGATCCTGCC–3′ (RH11 derivates) 5′–CTGCTGCCGTCCTTGGATGT–3′ (Gia2150c) Equal mix of 5′–AGTCGAACCCTGATTCTCCGCCAGG–3′ and 5′–AGTCAAACCCTGATCCTCCGCCAGG–3′ and 5′–AGTCGAACCCTGATTCTCCGTCAGG–3′ (RH4–derivates)	[61]

**Table 2:** Frequency of enteric protists in animals residing in Lisbon Zoo, Maia Zoo and Pedagogical Farm of Canelas, Portugal.

Parasite	Positive Samples (*n*)	Frequency (*%*)	95% Confidence Interval
*B. coli*	6	2.9	0.01–0.06
*Blastocystis* sp.	24	11.6	0.08–0.17
*Cryptosporidium* spp.	1	0.5	0.00–0.03
*Eimeria* spp.	1	0.5	0.00–0.03
*Giardia* spp.	1	0.5	0.00–0.03

**Table 3:** Host species and Genbank ID of *B. coli* sequences generated in this study. Single nucleotide polymorphisms of *B. coli* sequences obtained at the genetic marker used in the present survey (ITS1–5.8s-rRNA–ITS2 region and the last 117 bp at the 3t’ end of the *SSU*-rRNA gene) are also shown.

Isolate	Genbank ID	Host Species	Variant	Reference	Stretch	SNPs
ZL30	OM349074	*Sus scrofa domesticus*	B	JQ073362	8–506	A8T, T399C
ZL32	OM349075	*Sus scrofa domesticus*	B	JQ073366	18–505	T479C, G505A
ZL46	OM349076	*Tayassu tajacu*	B	JQ073366	11–507	T470C, G505A, A506T, T507G
ZM74	OR987597	*Lutra lutra*	B	JQ073366	120–499	T469A
Q28	OR987497	*Eublepharis macularis*	A	JQ073359	145–503	T246C
Q36	OR987498	*Sus scrofa domesticus*	B	JQ073362	76–479	T399C

**Table 4:** Host species and Genbank ID of *Blastocystis* sp. positive samples. Single nucleotide polymorphisms of *Blastocystis* sp. sequences obtained at the *SSU*-rRNA genetic marker used in the present survey are also shown. Note that for isolates whose subtype has not been identified, SNPs were not determined (isolates from reptilian sources and isolate ZM60).

Isolate	Genbank ID	Host Species	*Blastocystis* sp. STs	Reference	Stretch	SNPs
ZL1	OR987545	*Gorilla gorilla*	ST1	MT898451	31–584	A71G, T225G, G226T, G382A, A475T, C477T, A478C
ZL4	OR987546	*Aepyceros melampus*	ST24	MW887928	28–585	G113T
ZL14	OR987547	*Addax nasomaculatus*	ST10a	MZ265404	22–593	-
ZL40	OR987548	*Colobus guereza*	ST1	MT898451	32–579	A71G, T225G, G226T, C477T, A478C
ZL57	OR987549	*Choeropsis liberiensis*	ST3	AB091234	2–554	A104T, A150G, A238T,
ZL60	OR987550	*Trachypithecus auratus*	ST13	KC148209	10–586	G119T, A121C, A164C, T165C, G166A, T173C, G436C, C502T, T507C
ZL68	OR987551	*Macaca fuscata*	ST1	MT898451	22–594	A71G, G129A, T225G, G226T, C477T, A478C
ZL69	OR987552	*Cercopithecus nictians*	ST1	MT898451	29–583	A71G, T225G, G226T, C477T, A478C
ZM31	OR989903	*Rhea americana*	ST13	KC148209	37–573	C153A
ZM33	OR989904	*Python regius*	-	-	-	-
ZM45	OR989905	*Python bivittatus*	-	-	-	-
ZM46	OR989906	*Tiliqua scincoides*	-	-	-	-
ZM47	OR989907	*Pogona vitticeps*	-	-	-	-
ZM48	OR989908	*Python bivittatus*	-	-	-	-
ZM49	OR989909	*Python bivittatus*	-	-	-	-
ZM52	OR98910	*Iguana iguana*	-	-	-	-
ZM60	OR989911	*Crax rubra*	-	-	-	-
ZM63	OR989912	*Zonosaurus maximus*	-	-	-	-
ZM64	OR989913	*Morelia spilota variegate*	-	-	-	-
ZM67	OR989914	*Python reticulatus*	-	-	-	-
ZM70	OR989915	*Cercopithecus ascanius*	ST1	MT898451	14–596	A71G, T225G, G226T, C477T, A478C,
ZM72	OR989916	*Chlorocebus pygerythrus*	ST2	AB070997	120–520	T123G, T126G, C135T, T140G, G142A, C159T, C165T, T166A, A170C, T173G, C183T, T187G, T228G, G229T, T268A, A477G, C505T,
Q32	OR977104	*Sus scrofa domesticus*	ST5	AB070998	28–497	C176A, A262G
Q33	OR987485	*Sus scrofa domesticus*	ST5	AB070998	23–496	C176A, A262G

**Table 5:** Single nucleotide polymorphisms of the *Cryptosporidium* spp. sequence obtained at the *SSU*-rRNA genetic marker used in the present survey.

Isolate	Genbank ID	Host Species	Reference	Stretch	SNPs
Q16	OR987500	*Equus ferus caballus*	MT026934	375–693	A381C, G466A, G499T, T577G, G615A, C630A, A654G, C677G, C689A

**Table 6:** Single nucleotide polymorphisms of the *Eimeria* spp. sequence obtained at the *SSU*-rRNA genetic marker used in the present survey.

Isolate	Genbank ID	Host Species	Reference	Stretch	SNPs
ZM26	OR987677	*Pavo cristatus*	HG793039	521–859	G633A; T639C; C646T; A679T; A688G; G689A; T717A; G718A; T719C; T768C

**Table 7:** Single nucleotide polymorphisms of the *Giardia* spp. sequence obtained at the *SSU*-rRNA genetic marker used in the present survey.

**Isolate**	**Genbank ID**	**Host Species**	**Reference**	**Stretch**	**SNPs**
ZM40	OR991119	*Pogona vitticeps*	AF113895	76–264	G257A

## Data Availability

Data presented in this study are available on request from the corresponding author.

## Supplementary Materials

The following supporting information can be downloaded at: https://www.mdpi.com/article/10.3390/pathogens13100874/s1, Table S1: Number of individuals by family in the total number of samples; Table S2: Number of individuals by family in samples collected at the Lisbon Zoo; Table S3: Number of individuals by family in samples collected at the Maia Zoo; Table S4: Number of individuals by family in samples collected at the Pedagogical Farm of Canelas; Figure S1: Visual representation of collected samples by zoological garden of origin.; Table S5: PCR cycling conditions used for the molecular identification of the protists covered in this work.
