# Supplementary material for: Multicentric Study on Enteric Protists Occurrence in Zoological Parks in Portugal
Source: Pathogens. 2024 Oct 6;13(10):874. doi: 10.3390/pathogens13100874 (PMC11509964; doi:10.3390/pathogens13100874)
Supplement: Supplementary file 1 [file pathogens-13-00874-s001.zip › pathogens-3216349-supplementary.pdf]

Supplementary Materials

Families represented in this work

From the Lisbon Zoo, faecal samples (*n*=81) were collected from 58 species of mammals from 20 distinct families (Ailuridae, Bovidae, Callitrichidae, Camelidae, Canidae, Cebidae, Cercopithecinae, Cervidae, Delphinidae, Equidae, Felidae, Giraffidae, Hippopotomidae, Hominidae, Lemuridae, Macropodidae, Pharcolarctidae, Rhinocerotoidae, Suidae and Tayassuidae). Samples were collected between February and April 2021.

From the Maia Zoo, faecal samples (*n*=76) were collected from 61 species of mammals from 39 distinct families (Agamidae, Ambystomatidae, Anatidae, Anguidae, Boidae, Bovidae, Callitrichidae, Camelidae, Cebidae, Cercopithecidae, Cervidae, Colubridae, Cracidae, Dasypodidae, Didelphidae, Dromaiidae, Equidae, Estrildidae, Felidae, Gerrhosauridae, Gruidae, Herpestidae, Hylidae, Hylobatidae, Hystricidae, Iguanidae, Lemuridae, Macropodidae, Mustelidae, Phasianidae, Phoenicopteridae, Psittacidae, Psittaculidae, Pythonidae, Rheidae, Scincidae, Teiidae, Testudinidae and Ursidae). The samples were collected in September 2023.

From the Pedagogical Farm of Canelas, faecal samples (*n*=50) were collected from 12 species, from 10 distinct families (Agamidae, Anatidae, Bovidae, Equidae, Gekkonidae, Leporidae, Phasianidae, Spirostreptidae, Suidae and Varanidae). The samples were collected in September 2023. Number of individuals per family is described in Tables 1, 2, 3 and 4.

Table S1. Number of individuals by family in the total number of samples.

Family	Lisbon Zoo (*n*)	Maia Zoo (*n*)	Pedagogical Farm of Canelas (*n*)	Total (*n*)
**Agamidae**	0	3	2	5
**Ailuridae**	1	0	0	1
**Ambystomatidae**	0	3	0	3
**Anatidae**	0	2	3	5
**Anguidae**	0	1	0	1
**Boidae**	0	1	0	1
**Bovidae**	24	1	6	31
**Callitrichidae**	1	3	0	4
**Camelidae**	2	1	0	3
**Canidae**	2	0	0	2
**Cebidae**	2	1	0	3
**Cercopithecinae**	8	3	0	11
**Cervidae**	3	1	0	4
**Colubridae**	0	5	0	5
**Cracidae**	0	2	0	2
**Dasypodidae**	0	1	0	1
**Delphinidae**	1	0	0	1
**Didelphidae**	0	1	0	1
**Dromaiidae**	0	1	0	1
**Equidae**	1	1	25	27
**Estrildidae**	0	1	0	1
**Felidae**	10	6	0	16
**Gekkonidae**	0	0	1	1
**Gerrhosauridae**	0	1	0	1
**Giraffidae**	2	0	0	2
**Gruidae**	0	2	0	2
**Herpestidae**	0	1	0	1
**Hippopotomidae**	2	0	0	2
**Hominidae**	4	0	0	4
**Hylidae**	0	1	0	1
**Hylobatidae**	0	2	0	2
**Hystricidae**	0	1	0	1
**Iguanidae**	0	2	0	2
**Lemuridae**	5	3	0	8
**Leporidae**	0	0	2	2

**Macropodidae**	2	1	0	3
**Mustelidae**	0	2	0	2
**Pharcolarctidae**	3	0	0	3
**Phasianidae**	0	1	6	7
**Phoenicopteridae**	0	1	0	1
**Psittacidae**	0	3	0	3
**Psittaculidae**	0	2	0	2
**Pythonidae**	0	7	0	7
**Rheidae**	0	1	0	1
**Rhinocerotoidae**	4	0	0	4
**Scincidae**	0	2	0	2
**Spirostreptidae**	0	0	1	1
**Suidae**	3	0	3	6
**Tayassuidae**	1	0	0	1
**Teiidae**	0	1	0	1
**Testudinidae**	0	3	0	3
**Ursidae**	0	1	0	1
**Varanidae**	0	0	1	1

Table S2. Number of individuals by family in samples collected at the Lisbon Zoo.

Family	Number of samples (*n*)
**Hominidae**	4
**Delphinidae**	1
**Ailuridae**	1
**Felidae**	10
**Canidae**	2
**Pharcolartidae**	3
**Macropodidae**	2
**Hippopotomidae**	2
**Cervidae**	3
**Camelidae**	2
**Suidae**	3
**Giraffidae**	2
**Bovidae**	24
**Rhinocerotoidae**	4
**Equidae**	1
**Lemuridae**	5
**Cercopithecinae**	8

**Callitrichidae**	1
**Cebidae**	2
**Tayassuidae**	1

Table S3. Number of individuals by family in samples collected at the Maia Zoo.

Family	Number of samples (*n*)
**Felidae**	6
**Ursidae**	1
**Bovidae**	1
**Testudinidae**	3
**Cervidae**	1
**Equidae**	1
**Camelidae**	1
**Macropodidae**	1
**Hystricidae**	1
**Dasypodidae**	1
**Cercopithecidae**	3
**Lemuridae**	3
**Hylobatidae**	2
**Cebidae**	1
**Callitrichidae**	3
**Dromaiidae**	1
**Anatidae**	2
**Psittacidae**	3
**Psittaculidae**	2
**Phasianidae**	1
**Phoenicopteridae**	1
**Rheidae**	1
**Cracidae**	2
**Gruidae**	2
**Estrildidae**	1
**Hylidae**	1
**Ambystomatidae**	3
**Didelphidae**	1
**Herpestidae**	1
**Mustelidae**	2
**Gerrhosauridae**	1
**Pythonidae**	7

**Iguanidae**	2
**Colubridae**	5
**Scincidae**	2
**Agamidae**	3
**Boidae**	1
**Teiidae**	1
**Anguidae**	1

Table S4. Number of individuals by family in samples collected at the Pedagogical Farm of Canelas.

Family	Number of samples (*n*)
**Equidae**	25
**Suidae**	3
**Bovidae**	6
**Agamidae**	2
**Gekkonidae**	1
**Varanidae**	1
**Spirostreptidae**	1
**Phasianidae**	6
**Anatidae**	3
**Leporidae**	2

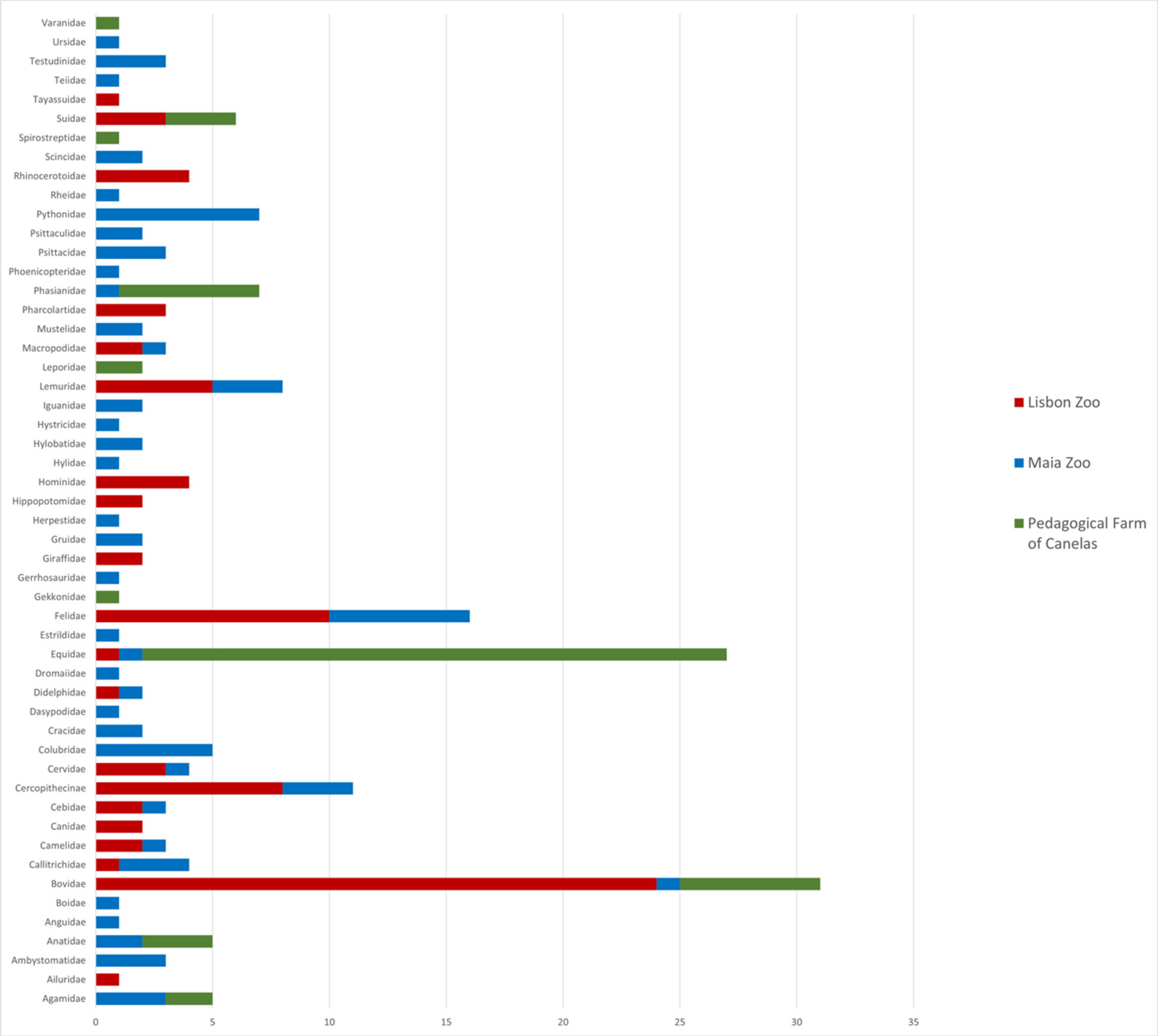

Figure S1. Visual representation of collected samples by zoological garden of origin.

PCR-cycling conditions

Table S5. PCR cycling conditions used for the molecular identification of the protists covered in this work.

		Temperature and time						
**Target organism**	**Locus**	**Initial denaturation**	**Denaturation**	**Annealing**	**Extension**	**No. cycles**	**Final extension**	**Reference**
B. coli	*SSU*-rRNA	95 °C – 5 min	94°C – 2 s	60 °C – 5 s	72 °C – 5 s	40	72 °C – 10 min	[56]
**Blastocystis sp.**	*SSU*-rRNA	95 °C – 5 min	94°C – 2 s	59 °C – 5 s	72 °C – 5 s	40	72 °C – 10 min	[57]
***Cryptosporidium* spp.**	*SSU*-rRNA	95 °C – 5 min	94°C – 2 s	50 °C – 5 s	72 °C – 5 s	40	72 °C – 10 min	[58]
	*gp*60	95 °C – 5 min	94°C – 2 s	58/55 °C – 5 s	72 °C – 5 s	35	72 °C – 10 min	[59]
***Eimeria* spp.**	*SSU*-rRNA	95 °C – 5 min	94°C – 2 s	56 °C – 5 s	72 °C – 5 s	40	72 °C – 10 min	[60]
***Giardia* spp.**	*SSU*-rRNA	95 °C – 5 min	94°C – 2 s	55/60 °C – 5 s	72 °C – 5 s	40	72 °C – 10 min	[61]

Results by zoological garden

From the 81 stool samples collected in the Lisbon Zoo, infection by GI protists was detected in 11 samples (13.6%, 11/81, 95% CI: 0.07–0.23). *Balantioides coli* was detected in three samples (3.7%, 3/81, 95% CI: 0.01–0.10), while *Blastocystis* sp. accounted for the other eight positive samples (9.9%, 8/81, 95% CI: 0.04–0.19). *Balantioides coli* was identified in two Vietnamese pot-bellied pigs (*Sus scrofa domesticus*) and a peccary (*Tayassu pecari*), while *Blastocystis* sp. was found in a gorilla (*Gorilla gorilla*), an impala (*Aepyceros melampus*), an addax (*Addax nasomaculatus*), a mantled guereza (*Colobus guereza*), a pygmy hippopotamus (*Choeropsis liberiensis*), an East Javan langur (*Trachypithecus auratus*), a Japanese macaque (*Macaca fuscata*) and a greater spot-nosed monkey (*Cercopithecus nictians*). No samples collected in the Lisbon Zoo tested positive for *Cryptosporidium* spp., *Eimeria* spp. and *Giardia* spp.

From the 76 stool samples collected in Maia Zoo, infection by GI protists was detected in 17 samples (22.4%, 17/76, CI: 0.14–0.33). *Blastocystis* sp. was the most prevalent protist species found (18.4%, 14/76; 95% CI: 0.10-0.29) followed by *B. coli*, *Eimeria* spp. and *Giardia* spp. (1.3%, 1/76; 95% CI: 0.00–0.07 each). The samples positive for *B. coli*, *Eimeria* spp. and *Giardia* spp. were isolated from an otter (*Lutra lutra*), a peacock (*Pavo cristatus*) and a central bearded dragon (*Pogona vitticeps*), respectively, while *Blastocystis* sequences were isolated from a greater rhea (*Rhea americana*), a royal python (*Python regius*), three Burmese pythons (*Python bivittatus*), a blue-tongued skink (*Tiliqua scincoides*), a central bearded dragon (*Pogona vitticeps*), an Iguana (*Iguana iguana*), a great curassow (*Crax rubra*), a Southeastern girdled lizard (*Zonossaurus maximus*), a Darwin carpet python (*Morelia spiglota variegate*), a reticulated python (*Python reticulatus*), a red-tailed monkey (*Cercopithecus ascanius*) and a vervet monkey (*Chlorocebus pygerythrus*). No sample collected in the Maia Zoo tested positive for *Cryptosporidium* spp. This location was the only one that presented a symptomatic case (ZM74) in which the individual in question, *Lutra lutra*, showed clinical signs of a GI infection (diarrhoea) and was quarantined.

From the total 50 stool samples collected in the Pedagogical Farm of Canelas, infection by GI protists was detected in five samples (10.0%, 5/50, CI: 0.03–0.22). Detection of *B. coli* and *Blastocystis* sp. occurred in two samples each (4.0%, 2/50, 95% CI: 0.02–0.19). *Cryptosporidium* spp. only accounted for one positive sample (2.0%, 1/50, 95% CI: 0.00–0.14). This sample positive for *Cryptosporidium* spp. was also positive when testing for the *gp*60 gene. Detection of *B. coli* occurred in a Leopard Gecko (*Eublepharis macularius*) and in a pig (*Sus scrofa domesticus*), while detection of *Blastocystis* sp. occurred only in pigs (*Sus scrofa domesticus*). The only positive sample of *Cryptosporidium* originated from a horse (*Equus ferus caballus*). No sample collected in the Pedagogical Farm of Canelas tested positive for *Eimeria* spp. and *Giardia* spp.
